# Weight matters: Higher BMI is associated with changes in the brain connectome in health and depression

**DOI:** 10.1016/j.nsa.2026.106984

**Published:** 2026-02-13

**Authors:** Aicha Bouzouina, Marius Gruber, Tong Zhao, Carmen Schiweck, Mareike Aichholzer, Carmen Uckermark, Luise V. Claaß, Frederike Stein, Nils R. Winter, Susanne Meinert, Kira Flinkenflügel, Dominik Grotegerd, Janik Goltermann, Tiana Borgers, Elisabeth Leehr, Linda Bonnekoh, Florian Thomas-Odenthal, Marc Pawlitzki, Paula Usemann, Lea Teutenberg, Igor Nenadić, Benjamin Straube, Nina Alexander, Hamidreza Jamalabadi, Andreas Jansen, Nils Opel, Tim Hahn, Andreas Reif, Tilo Kircher, Udo Dannlowski, Jonathan Repple, Sharmili Edwin Thanarajah

**Affiliations:** a Department of Psychiatry, Psychosomatic Medicine and Psychotherapy, University Medical Centre Frankfurt, Frankfurt am Main, Germany; b Biological Sciences, Goethe University Frankfurt, Frankfurt am Main, Germany; c Department of Psychiatry, Jena University Hospital/Friedrich-Schiller-University Jena, Jena, Germany; d Department of Psychiatry and Psychotherapy, University of Marburg, Marburg, Germany; e Institute for Translational Psychiatry, University of Münster, Münster, Germany; f Institute for Translational Neuroscience, University of Münster, Germany; g Department of Child and Adolescent Psychiatry, Psychosomatics and Psychotherapy, University of Münster, Germany; h Centre for Mind, Brain and Behaviour (CBMM), University of Marburg, Marburg, Germany; i Department of Neurology, Medical Faculty and University Hospital Düsseldorf, Heinrich Heine University, Düsseldorf, Germany; j Core Facility Brain Imaging, Faculty of Medicine, Philipps-University Marburg, Marburg, Germany; k Max Planck-Institute for Metabolism Research, Cologne, Germany; l Fraunhofer Institute for Translational Medicine and Pharmacology, Frankfurt, Germany; m Goethe University Frankfurt, Cooperative Brain Imaging Center (CoBIC), Frankfurt am Main, Germany

**Keywords:** BMI, Overweight, Connectome, White matter, Depression

## Abstract

Overweight and obesity are bidirectionally associated with major depressive disorder (MDD). However, the underlying mechanisms remain unknown. Here, we investigated whether the body mass index (BMI) is associated with alterations in the structural brain connectome of healthy participants and MDD patients and if these changes in connectivity are associated with clinical outcomes. We analyzed the association of BMI with structural brain connectivity in 746 MDD patients and 852 healthy controls from the Marburg-Münster Affective Disorders Cohort Study (MACS). The structural connectome was reconstructed using tractography in diffusion-weighted magnetic resonance imaging data. Associations between BMI brain connectivity were examined using network-based statistics (NBS). NBS identified a subnetwork of the brain connectome associated with BMI (*F*-threshold = 4.0, *p*_FWE_ < 0.05). The number of streamlines within this network were positively correlated with BMI (*β* = 56.122, SE = 5.50, *t* = 10.204, *p* < 0.001, R^2^ = 0.206), suggesting that an increase in BMI is linked to enhanced connectivity within the network. This association did not differ between healthy controls and MDD patients. BMI was further associated with depression severity (τ_BDI_ = 0.052, *p* = 0.002) and anhedonia (τ_SHAPS-D_ = 0.035, *p* = 0.034) across all participants, independent of diagnostic status. However, after controlling for BMI, connectivity within the BMI-associated subnetwork was not related to depression severity or anhedonia, suggesting that BMI-related brain connectivity alterations do not independently explain clinical symptom severity. Our findings reveal an association between BMI and structural brain connectivity, both in healthy controls and MDD patients. These findings indicate that increased body weight has a significant association with the brain structural connectome.

## 1 Introduction

Major Depressive Disorder (MDD) and obesity are leading causes of disability worldwide, with significant impact on personal well-being and public health (GBD, 2019 Mental Disorders Collaborators, 2022). The increasing prevalence of both conditions is alarming, given the bidirectional relationship: obesity increases the risk of being diagnosed with depression, and depression increases susceptibility to obesity (Quek et al., 2017; Holt et al., 2014; Mezuk et al., 2008; Milaneschi et al., 2019). This comorbidity is linked to worse disease outcomes and reduced treatment efficacy (Kraus et al., 2023; Kloiber et al., 2007), yet the mechanisms behind this association remain unclear.

Alterations in brain structure and function are consistently observed in both MDD and obesity (Zhang et al., 2023). Elevated body weight, typically quantified by the body mass index (BMI), has been linked to widespread brain structural alterations (Sochacka et al., 2024; Stunkard et al., 2003). For instance, grey matter volume reductions of prefrontal and temporal cortices have been reported in MDD patients with elevated BMI, which were linked to severe disease outcomes (Hidese et al., 2018; Opel et al., 2015) and reduced treatment response to antidepressant medication (Kloiber et al., 2007) as well as electroconvulsive therapy (Opel et al., 2021a). In addition, both MDD and elevated BMI have been linked to alterations in subcortical structures (Opel et al., 2021b; Yao et al., 2020). Emerging evidence, however, suggests that the impact of excess body weight extends beyond single regions, affecting the overall structural brain connectome (Chen et al., 2018). The brain connectome is defined as a network consisting of nodes that represent the brain regions and the edges that represent white matter streamlines derived by tractography from diffusion-weighted imaging data (Van Den Heuvel and Sporns, 2013).

Given the increasing evidence linking both obesity and MDD with brain structural alterations, we hypothesized that 1.) BMI is associated with alterations in a subnetwork of the structural brain connectome across healthy controls (HC) and MDD 2.) diagnosis (HC vs. MDD) and BMI show an interaction on brain connectivity and 3.) BMI and connectivity within the BMI-associated network are linked to clinical symptom severity. To test this, we applied network-based statistics (NBS) on brain structural data from a large single-study cohort including clinically diagnosed MDD patients and HCs.

## 2 Methods and materials

### 2.1 Participants

Participants in this study were part of the Marburg-Münster Affective Disorders Cohort Study (MACS) and were recruited via newspaper advertisements or local psychiatric hospitals at two sites (University of Marburg and University of Münster); see Kircher et al. (2019) for a general study protocol and Vogelbacher et al. (2018) for a magnetic resonance imaging (MRI) quality assurance protocol. Study procedures were approved by the ethics committees of the faculties of the universities of Marburg and Münster, and a total of *N* = 2071 participants (aged 18-65) gave written informed consent prior to examination. The Structured Clinical Interview for Diagnostic and Statistical Manual of Mental Disorders-IV Text Revision (DSM-IV-TR) (SCID-I; (Wittchen et al., 1997)) was used by trained personnel to diagnose psychiatric disorders or the lack thereof. Patients were included in our analysis if they were diagnosed with MDD. HCs were included in the analysis in case they had no current or history of psychiatric or neurological diseases. None of the participants had a history of severe somatic disease according to the MACS inclusion criteria. Participants from the MACS were excluded from the present analysis if they were diagnosed with a primary psychiatric disorder other than MDD or if they had no available connectome and BMI data. This resulted in the exclusion of 473 participants and a final total sample size of *N* = 1598 (see Table 1 for details).

**Table 1:** Demographic and clinical characteristics of the sample.

Variable	HC (*n* = 852)	MDD (*n* = 746)	*p*-value
Sex (m/f)	302/550 (35:65%)	270/476 (36:64%)	0.794
Age (years)	34.4 ± 12.95	36.28 ± 13.13	0.004
BMI (kg/m^2^)	23.97 ± 3.9	25.24 ± 4.67	<0.001
SHAPS-D	0.65 ± 1.23	3.34 ± 3.41	<0.001
BDI	4.11 ± 4.27	17.17 ± 10.97	<0.001
Age of Onset	–	25.50 ± 12.43	
Depressive episodes	–	3.60 ± 5.89	
Hospitalization	–	1.48 ± 1.90	
Medical load index	–	1.30 ± 1.46	

Note. Mean values and standard deviations are shown, except for sex. Test statistics and *p* values were derived from ANOVA or χ^2^ tests. BDI-I, Beck’s Depression Inventory (total score); SHAPS-D, Snaith-Hamiliton Pleasure Scale (total score); HC, healthy controls; MDD, Major Depressive Disorder patients.

### 2.2 Clinical outcomes

The Beck’s Depression Inventory (BDI-I) was used to assess the severity of the participants’ depression. It is widely used in both clinical and research settings and consists of 21 multiple-choice questions, each corresponding to a specific depressive symptom (e.g., sadness, pessimism, lack of interest, fatigue, and alterations in sleep or appetite) (Beck et al., 1961). Respondents assess the intensity of each symptom, and the aggregate score serves as an indicator of the depression's severity, with a range from minimal to severe. A score of 0 to 13 typically indicates minimal depression, suggesting either no or mild symptoms. A score between 14 and 19 reflects mild depression, where symptoms are noticeable but do not significantly impair daily functioning. Scores from 20 to 28 indicate moderate depression, with more pronounced symptoms that may affect functioning. Finally, a score between 29 and 63 represents severe depression, characterized by debilitating symptoms that require urgent clinical intervention.

Additionally, the German version of the Snaith-Hamilton Pleasure Scale (SHAPS-D) was used to assess the participants’ anhedonia levels, which is considered to be one of the core symptoms of depression. Anhedonia, characterized by a reduced ability to experience pleasure or interest in typically enjoyable activities, has been shown to be closely linked to both the emotional and physiological aspects of depression (Öngün Yılmaz and Köse, 2021; Shomaker et al., 2012). Importantly, this symptom has been identified as particularly relevant in the context of the relationship between depression and body weight. Individuals experiencing higher levels of anhedonia may demonstrate alterations in eating behavior, physical activity, and overall lifestyle, all of which can influence or be influenced by the BMI (Ibrahim et al., 2016). Therefore, assessing anhedonia allows for a deeper understanding of how depressive symptoms might interact with BMI and contribute to changes in weight regulation and metabolism. The SHAPS-D contains 14 items encompassing four distinct domains of hedonic experience: engagement in interests and pastimes, social interactions, sensory perceptions, and culinary experiences (Franz et al., 1998). Each item consists of four response categories: Strongly Agree, Agree, Disagree, and Strongly Disagree. Both “Agree” responses were coded with a value of 0 and both “Disagree” responses received a score of 1. Total scores ranged from 0 to 14, with higher scores indicating a higher level of anhedonia (Franz et al., 1998; Snaith et al., 1995). The SHAPS-D is the standard questionnaire to assess anhedonia in clinical investigations, with a high reliability and validity, particularly in clinical populations.

### 2.3 MRI data acquisition

Two MR scanners with different hardware and software configurations were used in the MACS for data acquisition, located at the departments of Psychiatry at the University of Marburg and the University of Münster. These differences included scanner models, acquisition environments, and site-specific hardware and software settings. MRI acquisition protocols were harmonized across sites as far as possible, and data quality was continuously monitored using a standardized quality assurance framework (Vogelbacher et al., 2018).

To account for potential variability arising from these factors, scanner-site was included as a covariate in all connectome analysis and regression analysis in line with our previous analysis (Repple et al., 2020, 2023). See the Supplementary Information (SI 1) for further details regarding MRI parameters at both sites.

### 2.4 Anatomical connectome reconstruction

Detailed information regarding quality control and reconstruction of the anatomical connectome is provided in the Supplementary Materials (SI 2–4). In brief, white matter connectivity between 114 cortical brain regions, defined by the Cammoun subdivision of the Desikan–Killiany atlas (Suckling and Bullmore, 2004; Reference Manual NBS v1.2), was reconstructed using the Connectivity Analysis Toolbox (CATO; (De Lange et al., 2023)). Structural connectivity was derived from diffusion-weighted MRI data using deterministic tractography. We deliberately restricted the connectome reconstruction to cortical regions to maximize robustness and cross-site comparability in this large multi-center dataset. Cortical streamline estimates tend to be more reliable across scanners and tractography pipelines than connections involving small subcortical nuclei, which are particularly susceptible to partial-volume effects, registration inaccuracies, and tractography-related false positives (Repple et al., 2020, 2023; De Lange et al., 2023; Suckling and Bullmore, 2004). This limitation is especially relevant given the characteristics of the diffusion MRI protocol used in the MACS, which is a single-shell acquisition with comparatively low b-values and a limited number of diffusion encoding directions. Under these conditions, reliable reconstruction of subcortical and cortico–subcortical pathways is constrained, and reconstruction approaches are effectively limited to conventional DTI-based models and deterministic tractography. Given these methodological constraints and our primary aim to identify BMI-related alterations at the level of large-scale structural brain networks that are consistently reconstructable across participants, we focused on cortical connectome topology as a conservative and reproducible framework for network-based analyses in both HC and MDD patients.

For each participant, the resulting network was stored in a connectivity matrix, with rows and columns representing cortical regions (nodes) and matrix entries representing white matter connections (edges). Edge connectivity strength was quantified by the number of reconstructed streamlines between pairs of nodes. An edge was included if it contained at least three reconstructed streamlines, a threshold chosen to balance sensitivity and specificity of the resulting connectivity matrices (Zalesky et al., 2010; Xia et al., 2013).

### 2.5 Statistical analysis

#### 2.5.1 Network-based statistic

Brain structural networks associated with a specific effect were identified using network-based statistics (NBS); 23), implemented in MATLAB R2023b, with the number of streamlines (NOS) serving as a metric of structural connectivity. NBS detects a significant effect by implementing mass univariate testing at the edge level, which is adjusted for family-wise error (FWE). Consequently, FWE-corrected linear models are utilized to assess the relationships between e.g., BMI and edge-wise number of streamlines. Given the multi-center nature of the dataset, scanner-site was explicitly modeled as a nuisance covariate in all NBS analyses. By including scanner-site as a regressor, variance attributable to systematic differences between acquisition sites—such as scanner hardware, software versions, and acquisition environments—was removed prior to testing associations with BMI. After applying a predefined threshold, NBS identifies the largest subnetwork of interconnected supra-threshold edges. The significance of the identified network was evaluated via permutation testing involving 5000 permutations, in which BMI values were randomized while maintaining the constancy of other variables such as age, sex, group (HC vs. MDD), and scanner site (Münster vs. Marburg) (Suckling and Bullmore, 2004). For all obtained networks, we applied an NBS *F*-threshold of *F* = 4.0. We selected this threshold to ensure that each individual edge was associated with the effect of interest at *p* < 0.05, thus including effects of meaningful size only. To assess the robustness of the identified BMI-associated subnetwork with respect to the choice of the primary threshold, sensitivity analyses were conducted across a range of F-thresholds (F = 0.1–6.0), as recommended in the NBS reference documentation (Reference Manual NBS v1.2). Across this range, the core topology of the identified subnetwork remained stable, while variations in threshold primarily affected network extent, with lower thresholds yielding more extended and higher thresholds more focal subnetworks. The primary threshold in NBS determines the initial test statistic cut-off for identifying connections within the brain network associated with the effect of interest. However, it is important to note that it does not indicate the statistical significance of the identified network. The significance of the network is ultimately determined through the permutation test, which controls for the family-wise error rate across different thresholds. While the threshold selection remains arbitrary, the sensitivity to thresholds can provide insights into the nature of the effect being studied: Networks identified at low thresholds typically capture widespread effects, whereas networks identified at high thresholds typically capture focal effects (Zalesky et al., 2010). BrainNet Viewer (version 1.63) was used to generate network figures (Xia et al., 2013).

#### 2.5.2 Analysis of associations between BMI, the structural connectome, depression severity and anhedonia

To test our first hypothesis that BMI is associated with alterations in a subnetwork of the structural brain connectome across the whole sample, we employed NBS and linear regressions testing the association between BMI and structural brain connectome while correcting for age, sex, group, and scanner-site. Linear regression analyses were performed using RStudio (version 2023.12.0 + 369). Key R packages included psych (2.5.6), ggplot2 (4.0.1), car (3.1-3), dyplr (1.1.4), emmeans (2.0.1), lme4 (1.1-38), and tidyverse (2.0.0). Log-transformation was performed using the base R-function log1p(). To determine whether the participants' diagnoses influenced these associations, we included an interaction term for diagnosis (HC vs. MDD) x BMI. Subsequently, to examine whether connectivity within the BMI-associated network was related to clinical symptom severity beyond the effect of BMI itself, we employed linear regression models between BMI and BDI as well as SHAPS-D, correcting for age, sex, group, scanner-site, and BMI). This two-step approach allowed us to first identify structural brain networks associated with BMI and subsequently test whether alterations in these networks are associated with clinical symptom severity independent of BMI. This strategy is consistent with previous neuroimaging studies in depression that controlled for BMI when examining brain–symptom associations to disentangle effects related to body weight from those related to clinical outcomes (Opel et al., 2015; Schmaal et al., 2016).

## 3 Results

### 3.1 Higher BMI was associated with increased connectivity in a subnetwork of the brain connectome

**Fig. 1:**
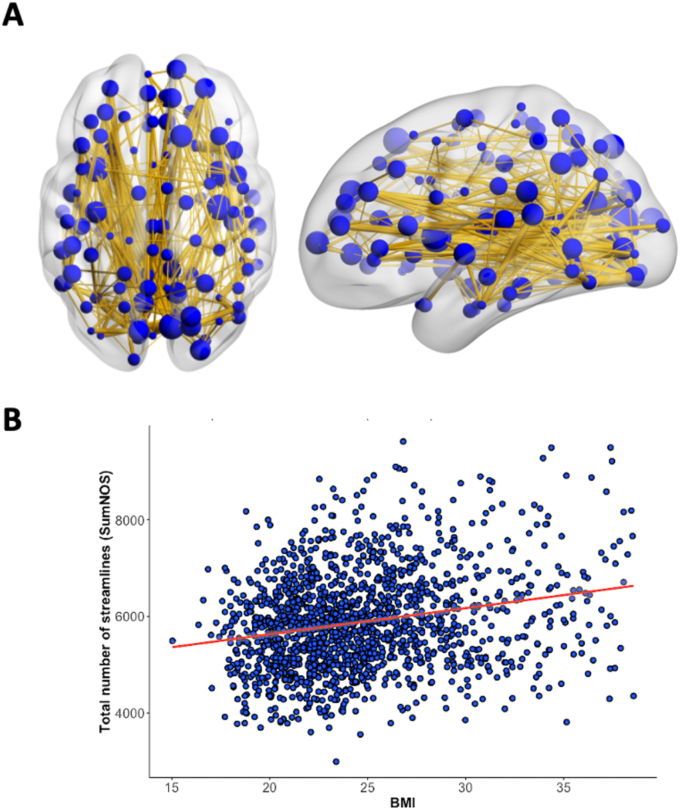
Subnetwork with a positive correlation between the total number of streamlines (NOS) and body mass index (BMI). (A) Shows a subnetwork based on a network-based-statistics analysis with a *p-*value (FWE-corrected) of *p* < 0.05 and a supra-threshold *F*-value *F* = 4.0. Edges (yellow) and nodes (blue) show a positive association between the brain connectivity based on the BMI across the entire sample, including major depressive disorder (MDD) patients and healthy control subjects (*p* < 0.001). Left panel: axial view. Right panel: sagittal view. (B) Scatter plot describing the association of SumNOS and BMI (r = 0.23, *p* < 0.001).

We tested whether BMI was associated with a subnetwork of the brain connectome (main effect). The NBS analysis revealed associations between the NOS and BMI in a subnetwork consisting of 309 edges (NBS *F*-threshold = 4.0) at a family-wise error-corrected significance level of *p*_FWE_ < 0.05 (see Fig. 1). Within this network, NOS was positively correlated with BMI (*β =* 56.122, SE = 5.50, *t* = 10.204, *p* < 0.001, R^2^ = 0.206), indicating that higher BMI is associated with increased connectivity. This association between BMI and NOS in the subnetwork was not significantly different between HCs and MDD patients (diagnosis∗BMI interaction, *β =* −17.25, SE = 10.56, *t* = −1.64, *p* = 0.101, R^2^ = 0.207). To test for a U-shaped relationship, we investigated the association between squared BMI and brain connectome, but no significant subnetwork was identified.

To further validate our findings, we analyzed connectivity matrices that were weighted by tract-wise average fractional anisotropy (FA). Using this measure of structural connectivity, we also found a BMI-related subnetwork (NBS F-threshold = 4.0), showing positive associations between FA and BMI (*β* = 0.192, SE = 0.022, *t* = 8.682, *p* < 0.001, R^2^ = 0.126) (see SI 5).

### 3.2 Increased connectivity in the BMI-associated network was not linked to symptom severity or higher anhedonia

**Fig. 2:**
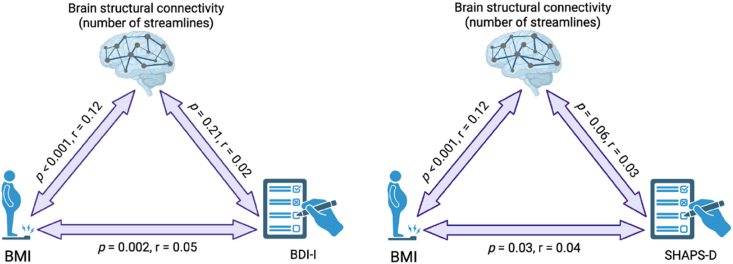
Triad analysis for the association between BDI-I/SHAPS-D total scores, BMI and structural brain connectivity. First, partial correlations were performed between the BDI-I/SHAPS-D total scores and BMI of all participants. Subsequently, partial correlations were calculated between BMI and the NOS of the identified BMI main effect subnetwork (brain structural connectivity). Lastly, partial correlations were conducted between the NOS and BDI-I/SHAPS-D total scores.

Next, we tested the association of BMI and NOS of the BMI-associated network with depression severity and anhedonia using partial correlations. Higher BMI was associated with higher depression severity (r_BMI,BDI_ = 0.052, *p* = 0.002) and anhedonia across all participants (r_BMI,SHAPS-D_ = 0.040, *p* = 0.032) (see Fig. 2). Within the BMI-associated network, NOS were not associated with depression severity (r_NOS,BDI_ = 0.021, *p* = 0.213). We found a significant association with NOS for anhedonia (r_NOS,SHAPS-D_ = 0.040, *p* = 0.037). However, after controlling for BMI, this relationship did not reach significance level (r_NOS,SHAPS-D_ = 0.031, *p* = 0.060).

## 4 Discussion

In a large bi-centric cohort study of patients with Major Depressive Disorder (MDD) as well as healthy controls (HCs), we identified a subnetwork within the structural brain connectome that was associated with body mass index (BMI), with higher BMI predicting increased connectivity. Across diagnostic groups, higher BMI was associated with higher levels of self-reported depression and anhedonia. However, contrary to our hypotheses, connectivity within the BMI-related subnetwork did not relate to symptom severity.

### 4.1 Elevated body weight is linked to increased connectivity within a subnetwork of the structural brain connectome

In this study, we investigated the brain structural connectome derived from diffusion-weighted imaging data comprising 114 nodes from cortical regions. We found a widespread subnetwork that was correlated with BMI. Connectivity within this subnetwork showed a positive association with BMI and showed no interaction with diagnosis, indicating that the association between BMI and brain connectivity goes beyond the effects of diagnosis. Overweight and obesity have been consistently linked to alterations in grey matter volume (GMV) and white matter integrity (Carbine et al., 2020): Elevated weight has been linked to GMV reduction, accelerated brain aging, and cognitive decline (Opel et al., 2021b; Li et al., 2022; Khatun et al., 2023; Gómez-Apo et al., 2021). On the other hand, some studies have also observed increased cortical thickness in specific brain regions with increased body weight, suggesting a complex structural reorganization (Zhang et al., 2022; Kim et al., 2015). Despite these findings, studies examining the relationship between BMI and structural brain connectome alterations using a network-based approach remain limited. To date, only one study using a small sample of 20 obese and 30 normal weight participants (Chen et al., 2018) identified that obesity was associated with an altered brain connectome, showing fewer network connections by using graph theoretical and NBS analysis. It is important to note that, in comparison to this study, we included BMI as a continuous variable, and only a minority of patients in our sample were severely obese. In our data, the relationship between BMI and brain connectome was not U-shaped. However, since the transition to obesity goes along with substantial changes in peripheral metabolism and inflammation (Dong et al., 2025), it is possible that brain connectome alteration in severely obese participants differs from that of those with overweight.

### 4.2 Connectivity strength of the BMI-associated network is not linked to symptom severity

In line with previous evidence, metabolic dysregulation has been constantly linked to depression and anhedonia in both human and animal models, with evidence supporting a bidirectional relationship. In rodents, obesity resulting from a high-fat diet has been demonstrated to induce anhedonia (Carr et al., 2023), while longitudinal human data indicate that elevated anhedonia increases the risk of future weight gain, suggesting a self-reinforcing cycle between reward dysfunction and obesity (Cho et al., 2018). The underlying mechanisms are still unclear. Anhedonia has been largely associated with alterations in brain reward circuitry, particularly involving subcortical regions such as the ventral striatum and basal ganglia. Rodent studies demonstrated that both diet-induced obesity and insulin receptor knock-out lead to impaired brain dopamine signaling (Kleinridders et al., 2015; Han et al., 2021). However, emerging evidence indicates that anhedonia reflects dysfunction within distributed cortico-subcortical networks rather than isolated regional abnormalities (Wang et al., 2024). Consistent with this view, recent functional MRI studies demonstrated changes in the overall brain functional connectome to be associated with anhedonia (Dan et al., 2024; Yang et al., 2024). In the present study, we could not find an association between connectivity within the BMI-associated structural subnetwork and depression severity or anhedonia after controlling for BMI. The interpretation of this null finding warrants caution, as the absence of a statistical association does not allow conclusions regarding the absence of an underlying effect and may reflect a combination of biological, methodological, and statistical factors. One relevant methodological consideration is that our structural connectome was restricted to cortical regions and did not include subcortical nodes that are central to reward processing. Given the prominent role of cortico–striatal and subcortico–subcortical circuits in motivational and hedonic processes, it is plausible that clinically relevant BMI-related alterations underlying anhedonia reside primarily within these pathways and were not captured by our cortical-only connectome. At the same time, previous studies—including work from our group (Repple et al., 2020; Gruber et al., 2023; Stein et al., 2024)—have demonstrated associations between cortical network measures and clinical symptoms, indicating that the exclusion of subcortical regions alone is unlikely to fully explain the present null result. Consequently, while our findings suggest that cortical structural network alterations associated with BMI do not independently explain symptom severity, they do not preclude a mechanistic role of subcortical reward circuitry in linking obesity to anhedonia. Future studies integrating cortico-subcortical connectivity, higher resolution diffusion imaging, and complementary functional approaches will be essential to more fully elucidate the neural mechanisms underlying the relationship between obesity and anhedonia.

### 4.3 Underlying biological mechanisms are multifactorial and need further exploration

Biological mechanisms linking brain connectome and BMI are multifaceted and remain to be thoroughly explored (García-García et al., 2022). Overweight and obesity can significantly impact brain structure and function through chronic low-grade inflammation that is associated with blood-brain-barrier dysfunction and neuroinflammation (Feng et al., 2024). Moreover, metabolic dysregulation with initial hyperinsulinemia and insulin resistance directly impacts neuronal signaling and astrocyte function. Particularly, the impact of insulin on myelin sheaths is relevant: During brain development, insulin has a promoting effect on myelin-producing oligodendrocytes (McMorris and Dubois‐Dalcq, 1988). Insulin resistance has been associated with microstructural changes and both increases and decreases in myelin water fraction (MWF) in middle-aged adults using neuroimaging techniques (O’Grady et al., 2019). This suggests that hyperinsulinemia, strongly associated with rising BMI, may promote oligodendrocyte activity in specific neural networks. However, this is only a speculation and needs further clarification in rodent studies. In addition, both obesity as well as hyperinsulinemia and hyperglycemia have been associated with substantial changes in the extracellular matrix that critically affects the brain connectome (Morawski et al., 2014; Rauch, 2004; Lau et al., 2013) by changing collagen- and elastin fiber integrity. For instance, Özkan et al. (2023) demonstrated in a rodent study that a high-fat/high-sucrose-induced obesity was associated with widespread non-functional angiogenesis, brain-blood-barrier leakage and fragmentation and accumulation of collagen and elastin.

### 4.4 Limitations

Due to the cross-sectional nature of our study, it is not possible to draw causal conclusions. Longitudinal and intervention studies are needed to investigate the effects of BMI on neural networks in more detail and examine the causal relationships between these components. Specifically, it would be valuable to investigate whether BMI-related connectivity changes could serve as predictors for future depressive symptoms. Another important limitation is that our NBS analysis does not include subcortical brain areas, such as the basal ganglia, which are particularly relevant in the context of anhedonia. This choice was motivated by the limited reliability of diffusion MRI–based tractography for reconstructing connections involving small subcortical nuclei, which are particularly susceptible to partial-volume effects, registration inaccuracies, and tractography-related false-positive connections (Jones et al., 2013; Maier-Hein et al., 2017). These limitations are especially relevant in multi-center datasets, where differences in acquisition protocols, scanner hardware, and reconstruction pipelines can further amplify variability in subcortical pathways (Fortin et al., 2017). Subcortical regions, such as the ventral striatum and nucleus accumbens, play a central role in reward processing and anhedonia, and it is therefore possible that clinically relevant BMI-related alterations involve cortico–subcortical circuits that are not adequately captured by a cortical-only atlas (Treadway and Zald, 2011; Der-Avakian and Markou, 2012). Future studies should therefore integrate improved subcortical parcellations and complementary imaging modalities, such as functional connectivity analyses, task-based reward paradigms, or higher-resolution diffusion protocols combined with advanced tractography approaches, to more directly investigate the involvement of subcortical reward circuitry in the relationship between BMI and depressive symptomatology. A further limitation of our study is the exclusive reliance on BMI to assess overweight and obesity. Although BMI remains a widely accepted standard, it does not accurately reflect fat distribution relevant to metabolic health. Metrics such as waist-to-hip ratio or body fat percentage measured through whole-body MRI or bioelectrical impedance scales could offer more comprehensive insights. For instance, the meta-analysis of Xu et al. (2011) confirmed that depression was more closely associated with abdominal obesity than general obesity. Lastly, the BDI and SHAPS-D questionnaires rely on self-reports and are not objective measures.

### 4.5 Conclusion

In conclusion, this study highlights that higher BMI is linked to widespread alterations in the brain structural connectome that go beyond diagnosis. Future interventional and longitudinal studies in humans and rodents are required to assess the causal influence of body weight and, in particular, fat mass on brain connectome alteration and unravel the underlying biological mechanisms to identify new targets for prevention and treatment.

## Author contributions

Conception and design: AB, MG, JR and SET.

Acquisition of the data: MG, FS, NRW, SM, KF, DG, JG, TB, EL, LB, FTO, MP, PU, LT, IN, BS, NA, HJ, AJ, NO, TH TK and UD.

Data preprocessing: MG.

Data analysis: AB, MG.

Data interpretation: AB, MG, TZ, CS, MA, CU, LVC, AR, JR, SET.

Administrative, technical, or material support: TK, UD, AR.

Supervision: JR and SET.

Funding acquisition: TK, UD, AR, JR and SET.

Writing – original draft: AB, MG, JR and SET.

Writing – review & editing: all authors.

All authors had full access to all the data in the study and had final responsibility for the decision to submit for publication.

## Funding

10.13039/100024877AB and SET were funded by the Leistungszentrum Innovative Therapeutics (TheraNova) funded by the Fraunhofer Society and the Hessian Ministry of Science and Art. SET was funded by the Bundesministerium für Bildung und Forschung (10.13039/501100002347BMBF, Federal Ministry of Education)- 01EO2102 INITIALISE Advanced Clinician Scientist Program and the REISS foundation. MA was funded by the 10.13039/501100001659Deutsche Forschungsgemeinschaft (10.13039/501100001659DFG, 10.13039/501100001659German Research Foundation) - 493624332 INDEEP Clinician Scientist Program and served on one advisory board from Janssen. This work is part of the LOEWE Center “DYNAMIC”, funded by the HMWK Hessen.

## Conflict of interest

MG has received remuneration from Janssen for consultancy services. JR received speaker’s honoraria from Janssen, Hexal, Neuraxpharm and Novartis. AR has received honoraria for lectures and/or advisory boards from Janssen, Boehringer Ingelheim, COMPASS, SAGE/Biogen, LivaNova, Medice, Shire/Takeda, MSD and cyclerion. Also, he has received research grants from Medicine and Janssen. MA served on one advisory board from Janssen. SET served on an advisory board of Johnson and Johnson. All other authors report no conflict of interest.
