# Supplementary material for: Weight matters: Higher BMI is associated with changes in the brain connectome in health and depression
Source: Neurosci Appl. 2026 Feb 13;5:106984. doi: 10.1016/j.nsa.2026.106984 (PMC13080653; doi:10.1016/j.nsa.2026.106984)
Supplement: Multimedia component 1 [file mmc1.pdf]

Supplementary Information

Supplementary information 1: MRI Data Acquisition

T1 and diffusion-weighted images (DWI) were obtained utilizing 3T whole-body MRI scanners (Marburg: Tim Trio, 12-channel head matrix Rx-coil, Siemens, Erlangen, Germany; Münster: Prisma, 20-channel head matrix Rx-coil, Siemens, Erlangen, Germany). The GRAPPA acceleration factor of two was implemented for both sequences at the two scanners. Acquisition of a high-resolution T1-weighted dataset was carried out using a 3D-MPRAGE-sequence (TE = 2.28 ms, TR = 2130 ms, TI = 900 ms) with an isotropic voxel size of 1 x 1 x 1 mm^3. DTI imaging involved fifty-six axial slices with no gap, utilizing an isotropic voxel size of 2.5 x 2.5 x 2.5 mm^3 (TE = 90 ms, TR = 7300 ms). The imaging protocol included five non-DW images (b = 0 s/mm^2) and 2 x 30 DW images with a b-value of 1000 s/mm^2. Standardization of imaging pulse sequence parameters was implemented across both sites to the maximum extent allowed by each platform.

Supplementary information 2: Preprocessing of Diffusion Weighted Images

Diffusion-weighted images (DWI) underwent realignment and correction for eddy currents and susceptibility distortions (1) using the eddy function implemented in FSL 6.0.1 (1,2). The utilization of diffusion tensor imaging involves the representation of the signal measured in a voxel through a single tensor that characterizes the diffusion signal with a designated diffusion direction per voxel. The CATO toolbox (3), utilized for the reconstruction of the anatomical connectome, employs the informed RESTORE algorithm (4,5) for tensor estimation and outlier identification and removal during the fitting process, thus mitigating the influence of physiological noise artifacts on the DTI modeling. White matter pathways were reconstructed based on the diffusion profiles using deterministic tractography, which initiated eight seeds per voxel and generated a

tractography streamline for each seed by tracking the primary diffusion direction from voxel to voxel. Termination criteria encompass reaching a voxel with a fractional anisotropy < 0.1, executing a sharp turn of > 45°, reaching a gray matter voxel, or exiting the brain mask (6).

Supplementary information 3: Anatomical connectome reconstruction

We utilized the publicly accessible CATO toolbox (3) for the reconstruction of the anatomical connectome. The process encompassed a series of specific steps. A network consisting of 114 brain regions was acquired, along with the reconstructed white matter streamlines connecting these regions for each individual. The identification of brain regions was conducted using FreeSurfer's Desikan-Killiany Atlas (7–9). In light of the compromised DWI signal-to-noise ratio in subcortical regions and their significant impact on network properties, a modified version of this atlas focusing solely on cortical regions was employed, as previously undertaken (10,11). For the reconstruction of streamlines, a deterministic streamline tractography approach based on the Fiber Assignment by Continuous Tracking (FACT) algorithm was implemented (12). The selection of this deterministic algorithm over more sophisticated diffusion direction reconstruction methods was based on its ability to strike a reasonable trade-off between false-negative and false-positive fiber reconstructions (13). The inclusion of connections between two nodes, i.e., brain regions, required a minimum of three reconstructed streamlines connecting them. This thresholding approach was adopted to achieve a balance between sensitivity and specificity in the resultant connectivity matrices (14,15). Each participant's network was ultimately represented in a connectivity matrix, where nodes were denoted by rows and columns, and edges, indicating connectivity strength measured as the number of reconstructed streamlines (NOS) between two nodes, were reflected in the matrix entries.

Supplementary information 4: Quality control procedure for connectivity matrices

In order to ensure the integrity of the connectivity matrices, we adhered to the methodology outlined in reference (16) and employed multiple criteria to detect anomalies within the matrices. Various factors were considered for identifying outliers, 1) including the mean number of streamlines, 2) the mean fractional anisotropy, 3) the mean occurrence of each participant's connections (low value if the participant has "odd" connections), and 4) the mean occurrence of each participant's interconnected brain regions (high value if the participant misses frequent connections). Quartiles (Q1, Q2, Q3) and the interquartile range (IQR=Q3-Q1) were calculated for each metric. A data point was classified as an outlier if its value fell below Q1-1.5*IQR or above Q3+1.5*IQR for any of the four metrics.

Supplementary information 5: BMI subnetwork reconstruction based on fractional anisotropy

We reconstructed the networks based on fractional anisotropy (FA) rather than the number of streamlines (NOS) to validate our findings of the BMI subnetwork. FA is a scalar value between 0 and 1 that describes the degree of anisotropy of a diffusion process. Average FA values for each tract identified through tractography were used in this analysis. Using this approach, we identified a BMI-related subnetwork (NBS F-threshold = 4.0), which revealed positive associations between FA and BMI (β = 0.192, SE = 0.022, t = 8.682, p < 0.001, R^2 = 0.126).

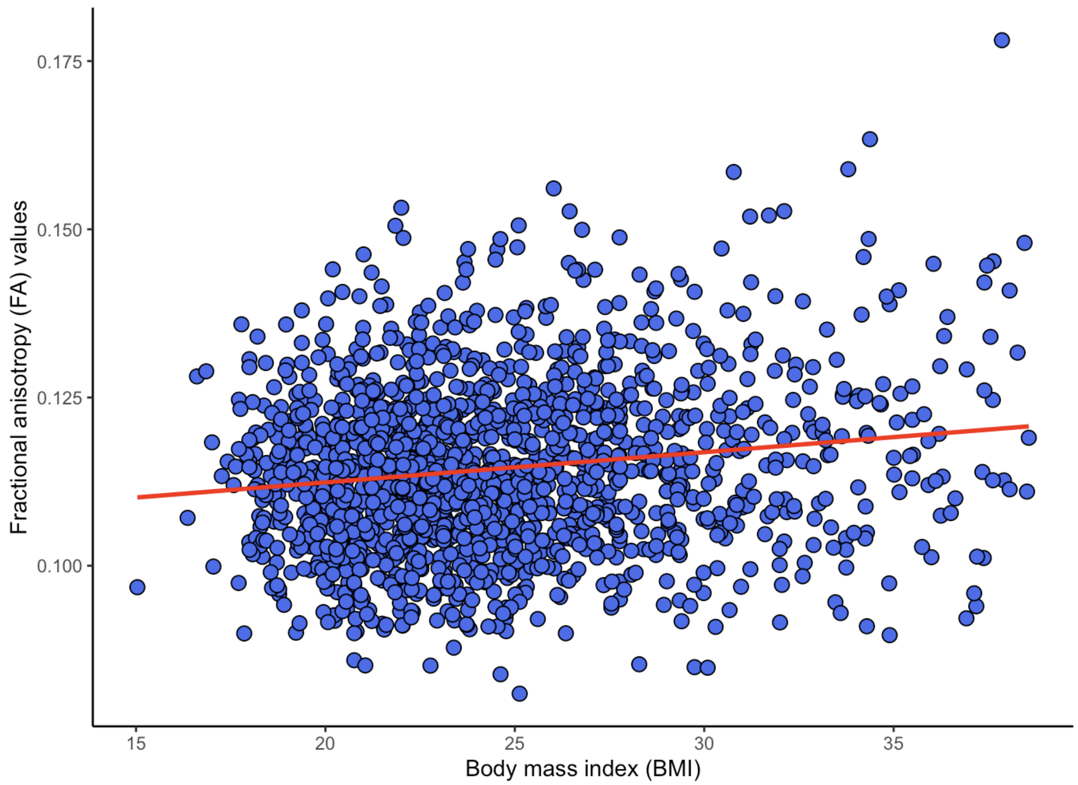

Supplementary information 6: Overlap of network edges

Supplementary table SI4: The table shows the overlap (in %) of edges that are included in the BMI associated subnetwork as well as the SHAPS-D associated subnetwork that were identified with Network-based statistics (NBS, *F*-threshold = 4.0). The overlap was calculated as the number of edges (= number of streamlines (NOS)) of a given network that are also part of a second network B divided by the total number of edges of network A multiplied by 100.

Network A	Network B	Overlap (%)	Mean overlap from permutations	LB 95% CI	UB 95% CI	*p*-value
SHAPS-D	BMI	9.71	5.24	2.29	8	0.029

Supplementary information 7: List of nodes and their connections in the BMI-associated network

supplementary table SI5: This table shows the pairs of brain regions connected by an edge that is part of the BMI associated subnetwork found with the NBS analysis. The NBS method was applied to the whole sample while correcting for age, sex and scanner site. The supra-threshold for the NBS analysis was set to a *F*-threshold of $F = 4.0$. The detected subnetwork consists of 309 edges and was found at a significance level of $p < .05$ using 5000 permutations.

**Brain region 1**		**Brain region 2**
left cuneus 1	↔	left fusiform 1
left entorhinal 1	↔	left fusiform 2
left bankssts 1	↔	left inferiorparietal 2
left fusiform 1	↔	left inferiortemporal 2
left entorhinal 1	↔	left isthmuscingulate 1
left inferiorparietal 1	↔	left isthmuscingulate 1
left inferiortemporal 2	↔	left lateraloccipital 1
left bankssts 1	↔	left lateralorbitofrontal 1
left fusiform 1	↔	left lateralorbitofrontal 1
left lateralorbitofrontal 1	↔	left lateralorbitofrontal 2
left cuneus 1	↔	left lingual 1

left fusiform 1	↔	left lingual 1
left fusiform 2	↔	left lingual 1
left cuneus 1	↔	left lingual 2
left fusiform 1	↔	left lingual 2
left fusiform 2	↔	left lingual 2
left lateralorbitofrontal 1	↔	left lingual 2
left lingual 1	↔	left lingual 2
left bankssts 1	↔	left middletemporal 1
left inferiorparietal 1	↔	left middletemporal 1
left inferiortemporal 2	↔	left middletemporal 1
left bankssts 1	↔	left middletemporal 2
left inferiortemporal 2	↔	left middletemporal 2
left fusiform 1	↔	left parahippocampal 1
left fusiform 2	↔	left parahippocampal 1
left inferiortemporal 1	↔	left parahippocampal 1
left isthmuscingulate 1	↔	left parahippocampal 1
left caudalanteriorcingulate 1	↔	left paracentral 1
left inferiortemporal 2	↔	left parsopercularis 1

left lateraloccipital 1	↔	left parsorbitalis 1
left lingual 2	↔	left parsorbitalis 1
left bankssts 1	↔	left parstriangularis 1
left lateraloccipital 2	↔	left parstriangularis 1
left lateralorbitofrontal 1	↔	left parstriangularis 1
left fusiform 1	↔	left pericalcarine 1
left lingual 1	↔	left pericalcarine 1
left lingual 2	↔	left pericalcarine 1
left parsorbitalis 1	↔	left pericalcarine 1
left bankssts 1	↔	left postcentral 1
left inferiorparietal 2	↔	left postcentral 1
left fusiform 1	↔	left postcentral 2
left inferiortemporal 1	↔	left postcentral 2
left inferiortemporal 1	↔	left postcentral 3
left middletemporal 1	↔	left postcentral 3
left isthmuscingulate 1	↔	left posteriorcingulate 1
left paracentral 1	↔	left posteriorcingulate 1
left caudalanteriorcingulate 1	↔	left precuneus 1

left inferiortemporal 2	↔	left precuneus1
left precentral 3	↔	left precuneus 1
left inferiorparietal 2	↔	left precuneus 2
left inferiortemporal 1	↔	left precuneus 2
left isthmuscingulate 1	↔	left precuneus 2
left lateralorbitofrontal 1	↔	left precuneus 2
left lingual 1	↔	left precuneus 2
left lingual 2	↔	left precuneus 2
left precuneus 1	↔	left rostralanteriorcingulate 1
left precuneus 1	↔	left rostralmiddlefrontal 1
left precuneus 2	↔	left rostralmiddlefrontal 1
left inferiortemporal 2	↔	left rostralmiddlefrontal 2
left lingual 1	↔	left rostralmiddlefrontal 2
left pericalcarine 1	↔	left rostralmiddlefrontal 2
left lingual 1	↔	left rostralmiddlefrontal 3
left medialorbitofrontal 1	↔	left rostralmiddlefrontal 3
left pericalcarine 1	↔	left rostralmiddlefrontal 3
left medialorbitofrontal 1	↔	left superiorfrontal 1

left parahippocampal 1	↔	left superiorfrontal 1
left rostralanteriorcingulate 1	↔	left superiorfrontal 1
left precuneus 2	↔	left superiorfrontal 2
left parstriangularis 1	↔	left superiorfrontal 4
left postcentral 2	↔	left superiorfrontal 4
left caudalmiddlefrontal 1	↔	left superiorparietal 1
left precuneus 2	↔	left superiorparietal 2
left inferiorparietal 1	↔	left superiorparietal 3
left inferiorparietal 2	↔	left superiorparietal 3
left lingual 1	↔	left superiorparietal 3
left postcentral 1	↔	left superiorparietal 3
left precuneus 2	↔	left superiorparietal 3
left rostralmiddlefrontal 1	↔	left superiorparietal 3
left inferiortemporal 1	↔	left superiortemporal 1
left inferiortemporal 2	↔	left superiortemporal 1
left middletemporal 1	↔	left superiortemporal 1
left middletemporal 2	↔	left superiortemporal 1
left inferiortemporal 1	↔	left superiortemporal 2

left lateralorbitofrontal 1	↔	left superiortemporal 2
left lateralorbitofrontal 2	↔	left superiortemporal 2
left medialorbitofrontal 1	↔	left superiortemporal 2
left fusiform 2	↔	left supramarginal 1
left inferiortemporal 2	↔	left supramarginal 1
left rostralmiddlefrontal 3	↔	left supramarginal 1
left superiortemporal 1	↔	left supramarginal 1
left bankssts 1	↔	left supramarginal 2
left inferiortemporal 1	↔	left supramarginal 2
left superiorparietal 3	↔	left supramarginal 2
left superiortemporal 1	↔	left supramarginal 2
left superiortemporal 2	↔	left temporalpole1
left parahippocampal 1	↔	left transversetemporal 1
left parsorbitalis 1	↔	left transversetemporal 1
left parstriangularis 1	↔	left transversetemporal 1
left inferiortemporal 1	↔	left insula 1
left lingual 2	↔	left insula 1
left parsopercularis 1	↔	left insula 1

left precuneus 2	↔	left insula 1
left superiorparietal 1	↔	left insula 1
left superiortemporal 2	↔	left insula 1
left transversetemporal 1	↔	left insula 1
left inferiortemporal 2	↔	left insula 2
left lateraloccipital 2	↔	left insula 2
left lateralorbitofrontal 2	↔	left insula 2
left lingual 1	↔	left insula 2
left lingual 2	↔	left insula 2
left medialorbitofrontal 1	↔	left insula 2
left parsorbitalis 1	↔	left insula 2
left pericalcarine 1	↔	left insula 2
left precentral 1	↔	left insula 2
left rostralmiddlefrontal 3	↔	left insula 2
left superiortemporal 2	↔	left insula 2
left transversetemporal 1	↔	left insula 2
left insula 1	↔	left insula 2
left caudalmiddlefrontal 1	↔	right caudalmiddlefrontal 1

left cuneus 1	↔	right cuneus 1
left entorhinal 1	↔	right cuneus 1
left fusiform 2	↔	right cuneus 1
left lateralorbitofrontal 1	↔	right cuneus 1
left lingual 1	↔	right cuneus 1
left medialorbitofrontal 1	↔	right cuneus 1
left parahippocampal 1	↔	right cuneus 1
left parsorbitalis 1	↔	right cuneus 1
left parstriangularis 1	↔	right cuneus 1
left pericalcarine 1	↔	right cuneus 1
left rostralmiddlefrontal 2	↔	right cuneus 1
left insula 1	↔	right cuneus 1
left insula 2	↔	right cuneus 1
left bankssts 1	↔	right cuneus 1
left precuneus 2	↔	right entorhinal 1
left cuneus 1	↔	right fusiform 2
left bankssts 1	↔	right inferiorparietal 1
left lateraloccipital 2	↔	right inferiorparietal 3

left fusiform 1	↔	right inferiortemporal 2
left inferiortemporal 1	↔	right inferiortemporal 2
left isthmuscingulate 1	↔	right isthmuscingulate 1
left lingual 1	↔	right isthmuscingulate 1
left pericalcarine 1	↔	right isthmuscingulate 1
left insula 2	↔	right isthmuscingulate 1
left insula 1	↔	right lateraloccipital 1
left insula 2	↔	right lateraloccipital 1
right bankssts 1	↔	right lateraloccipital 1
right cuneus 1	↔	right lateraloccipital 1
right inferiorparietal 3	↔	right lateraloccipital 1
right inferiortemporal 1	↔	right lateraloccipital 1
right inferiorparietal 2	↔	right lateraloccipital 2
right fusiform 1	↔	right lateraloccipital 3
right inferiortemporal 2	↔	right lateraloccipital 3
right lateraloccipital 2	↔	right lateralorbitofrontal 1
right inferiorparietal 3	↔	right lateralorbitofrontal 2
right lateralorbitofrontal 1	↔	right lateralorbitofrontal 2

right fusiform 1	↔	right lingual 1
right lingual 1	↔	right lingual 1
left pericalcarine 1	↔	right lingual 1
right fusiform 1	↔	right lingual 1
right fusiform 2	↔	right lingual 1
left fusiform 1	↔	right lingual 2
left lateraloccipital 2	↔	right lingual 2
left rostralanteriorcingulate 1	↔	right medialorbitofrontal 1
left rostralmiddlefrontal 2	↔	right medialorbitofrontal 1
left frontalpole 1	↔	right medialorbitofrontal 1
right fusiform 1	↔	right medialorbitofrontal 1
right bankssts 1	↔	right middletemporal 1
right bankssts 1	↔	right middletemporal 2
right lingual 1	↔	right middletemporal 2
right middletemporal 1	↔	right middletemporal 2
left superiorfrontal 2	↔	right paracentral 1
left caudalanteriorcingulate 1	↔	right parsopercularis 1
left caudalmiddlefrontal 1	↔	right parsopercularis 1

right caudalanteriorcingulate 1	↔	right parsopercularis 1
left caudalmiddlefrontal 1	↔	right parstriangularis 1
right inferiortemporal 2	↔	right parstriangularis 1
right lateralorbitofrontal 1	↔	right parstriangularis 1
right medialorbitofrontal 1	↔	right parstriangularis 1
left lateraloccipital 1	↔	right pericalcarine 1
left lateraloccipital 2	↔	right pericalcarine 1

References

1. Andersson JLR, Skare S. A Model-Based Method for Retrospective Correction of Geometric Distortions in Diffusion-Weighted EPI. NeuroImage. 2002 May;16(1):177–99.
2. Woolrich MW, Jbabdi S, Patenaude B, Chappell M, Makni S, Behrens T, et al. Bayesian analysis of neuroimaging data in FSL. NeuroImage. 2009 Mar;45(1):S173–86.
3. De Lange SC, Helwegen K, Van Den Heuvel MP. Structural and functional connectivity reconstruction with CATO - A Connectivity Analysis TOolbox. NeuroImage. 2023 Jun;273:120108.
4. Chang L, Jones DK, Pierpaoli C. RESTORE: Robust estimation of tensors by outlier rejection. Magn Reson Med. 2005 May;53(5):1088–95.
5. Chang L, Walker L, Pierpaoli C. Informed RESTORE: A method for robust estimation of diffusion tensor from low redundancy datasets in the presence of physiological noise artifacts.

Magn Reson Med. 2012 Nov;68(5):1654–63.

6. Van Den Heuvel MP, Sporns O, Collin G, Scheewe T, Mandl RCW, Cahn W, et al. Abnormal Rich Club Organization and Functional Brain Dynamics in Schizophrenia. JAMA Psychiatry. 2013 Aug 1;70(8):783.
7. Hagmann P, Cammoun L, Gigandet X, Meuli R, Honey CJ, Wedeen VJ, et al. Mapping the Structural Core of Human Cerebral Cortex. Friston KJ, editor. PLoS Biol. 2008 Jul 1;6(7):e159.
8. Cammoun L, Gigandet X, Meskaldji D, Thiran JP, Sporns O, Do KQ, et al. Mapping the human connectome at multiple scales with diffusion spectrum MRI. J Neurosci Methods. 2012 Jan;203(2):386–97.
9. Desikan RS, Ségonne F, Fischl B, Quinn BT, Dickerson BC, Blacker D, et al. An automated labeling system for subdividing the human cerebral cortex on MRI scans into gyral based regions of interest. NeuroImage. 2006 Jul;31(3):968–80.
10. Repple J, Mauritz M, Meinert S, De Lange SC, Grotegerd D, Opel N, et al. Severity of current depression and remission status are associated with structural connectome alterations in major depressive disorder. Mol Psychiatry. 2020 Jul;25(7):1550–8.
11. Gruber M, Mauritz M, Meinert S, Grotegerd D, De Lange SC, Grumbach P, et al. Cognitive performance and brain structural connectome alterations in major depressive disorder. Psychol Med. 2023 Oct;53(14):6611–22.
12. Mori S, Van Zijl PCM. Fiber tracking: principles and strategies – a technical review. NMR Biomed. 2002 Nov;15(7–8):468–80.
13. Sarwar T, Ramamohanarao K, Zalesky A. Mapping connectomes with diffusion MRI: deterministic or probabilistic tractography? Magn Reson Med. 2019 Feb;81(2):1368–84.
14. De Reus MA, Van Den Heuvel MP. Estimating false positives and negatives in brain networks. NeuroImage. 2013 Apr;70:402–9.
15. Zalesky A, Fornito A, Cocchi L, Gollo LL, Van Den Heuvel MP, Breakspear M. Connectome

sensitivity or specificity: which is more important? NeuroImage. 2016 Nov;142:407–20.

16. Van Den Heuvel MP, Scholtens LH, Van Der Burgh HK, Agosta F, Alloza C, Arango C, et al. 10Kin1day: A Bottom-Up Neuroimaging Initiative. Front Neurol. 2019 May 9;10:425.
